# Regulation of Circulating Sclerostin Levels by Sex Steroids in Women and in Men

**DOI:** 10.1002/jbmr.128

**Published:** 2010-05-17

**Authors:** Ulrike IL Mödder, Jackie A Clowes, Kelley Hoey, James M Peterson, Louise McCready, Merry Jo Oursler, B Lawrence Riggs, Sundeep Khosla

**Affiliations:** Endocrine Research Unit, College of Medicine, Mayo ClinicRochester, MN, USA

**Keywords:** ESTROGEN, TESTOSTERONE, SCLEROSTIN, BONE TURNOVER

## Abstract

Sex steroids are important regulators of bone turnover, but the mechanisms of their effects on bone remain unclear. Sclerostin is an inhibitor of Wnt signaling, and circulating estrogen (E) levels are inversely associated with sclerostin levels in postmenopausal women. To directly test for sex steroid regulation of sclerostin levels, we examined effects of E treatment of postmenopausal women or selective withdrawal of E versus testosterone (T) in elderly men on circulating sclerostin levels. E treatment of postmenopausal women (*n* = 17) for 4 weeks led to a 27% decrease in serum sclerostin levels [versus +1% in controls (*n* = 18), *p* < .001]. Similarly, in 59 elderly men, we eliminated endogenous E and T production and studied them under conditions of physiologic T and E replacement, and then following withdrawal of T or E, we found that E, but not T, prevented increases in sclerostin levels following induction of sex steroid deficiency. In both sexes, changes in sclerostin levels correlated with changes in bone-resorption, but not bone-formation, markers (*r* = 0.62, *p* < .001, and *r* = 0.33, *p* = .009, for correlations with changes in serum C-terminal telopeptide of type 1 collagen in the women and men, respectively). Our studies thus establish that in humans, circulating sclerostin levels are reduced by E but not by T. Moreover, consistent with recent data indicating important effects of Wnts on osteoclastic cells, our findings suggest that in humans, changes in sclerostin production may contribute to effects of E on bone resorption. © 2011 American Society for Bone and Mineral Research.

## Introduction

Both estrogen (E) and testosterone (T) are critical regulators of bone turnover, but the precise mechanisms by which these sex steroids have their effects on bone remain unclear. In women, E clearly reduces bone resorption,(1) and this effect is mediated by an inhibition of osteoclast development and activity, as well as increased osteoclast apoptosis (for review, see ref. 2). Effects of E on bone formation have been more difficult to define. E deficiency is associated with an increase in bone remodeling,(3) and the associated increase in bone resorption is accompanied by a coupled increase in bone formation at the tissue level.(2) However, at each basic multicellular unit (BMU) there remains a gap between bone resorption and bone formation, with formation unable to keep up with resorption, resulting in a net loss of bone.(2) By inference, therefore, sex steroid deficiency is associated with a defect in bone formation. Consistent with this, several studies now demonstrate that acute (3 to 4 weeks) E withdrawal(4) or treatment(5) results in a decrease and increase, respectively, in bone-formation markers, reflecting the underlying effects of E in maintaining bone formation at the cellular level. However, chronic E treatment of postmenopausal women is associated with reduced bone-resorption markers, leading, owing to the “coupling” of bone resorption and bone formation, to a reduction in bone-formation markers.(1,5) Studies in rats also have found that histologic bone-formation rates decrease early (at 5 days) following ovariectomy, but owing to the coupling with the increase in bone resorption, bone-formation rates are elevated 2 to 3 weeks following ovariectomy.(6) Thus, at the cellular level, E is important for the maintenance of bone formation in part due to a reduction in osteoblast apoptosis.(7)

Studies in men also have demonstrated a critical role for E in suppressing bone resorption and in maintaining bone formation.(8–10) Effects of E appear to be dominant over those of T, at least for bone resorption.(8) T effects on maintaining bone formation in humans appear to vary depending on the bone-formation marker used. We previously found that decreases in serum N-terminal propeptide of type 1 collagen (P1NP) levels following acute (3 weeks) E and T deficiency in men were prevented by E but not T, whereas both E and T were able to prevent decreases in serum osteocalcin (OCN) levels.(8)

The identification of the Wnt/β-catenin signaling pathway as a major regulator of bone mass(11,12) has led to considerable interest in potential crosstalk between this pathway and sex steroid signaling.(13) A major regulator of Wnt signaling is sclerostin, which is a secreted Wnt antagonist produced by osteocytes that regulates bone mass by binding to low-density lipoprotein receptor-related protein (LRP)5 and LRP6 to inhibit the canonical Wnt/β-catenin signaling pathway. The biologic importance of sclerostin in regulating bone mass in humans is highlighted by two genetic disorders associated with markedly increased bone mass: sclerosteosis and van Buchem disease.(14–17) These findings, combined with the demonstration that sclerostin-deficient mice have increased bone mass,(18) have led to the development of antisclerostin neutralizing antibodies as a novel anabolic treatment for osteoporosis.(19)

The recent development of immunoassays for sclerostin led to the observation by Mirza and colleagues(20) that postmenopausal women had higher serum sclerostin levels than premenopausal women, and in postmenopausal women, serum sclerostin levels were inversely associated with the circulating free estradiol (E_2_) index. These findings suggest that E may regulate sclerostin production; however, correlation does not prove causality, so in this study we tested the possible regulation by E of circulating sclerostin levels in women and in men. In addition, in the men, we also compared effects of E versus T on serum sclerostin levels and in both sexes related changes in sclerostin levels following hormonal manipulations to changes in bone turnover markers.

## Methods

### Study subjects and experimental protocol

#### Study A: Women

Serum samples for the sclerostin measurements were from a previous study from our group.(21) Briefly, 34 early postmenopausal women aged 40 to 65 years were randomized into an open-label, controlled study to receive E treatment or no treatment for 4 weeks. Menopausal status was defined by the absence of menses for more than 1 year in a woman over 50 years of age, and in women with previous hysterectomy or those under 50 years of age, an elevated value for serum follicle-stimulating hormone (FSH) was required. All women were healthy and had no clinically significant abnormalities in laboratory values and no diseases known to affect bone metabolism, and they were not taking any drug known to affect bone turnover. All subjects provided full informed consent, and the study was approved by the Mayo Clinic Institutional Review Board. Details regarding ensuring vitamin D sufficiency in the study subjects have been described previously.(21) Subjects randomized to E treatment (*n* = 17) received 17β-estradiol (17β-E_2_, 100 µg/day) by cutaneous patches (Vivelle, Novartis, East Hanover, NJ, USA) that were changed every 3 to 4 days for 4 weeks. Fasting (8 a.m.) serum samples were drawn at baseline and at 28 days of treatment (or no treatment in the control group). Serum samples were frozen and stored at −80°C, and only previously unthawed samples were used in all analyses in this study.

#### Study B: Men

The samples for this study were derived from a previously published study on the regulation of bone turnover by sex steroids in men.(10) Briefly, 59 elderly men aged 50 to 80 years were recruited for the study. As for study A, all men were healthy and had no clinically significant abnormalities in laboratory values, no diseases known to affect bone metabolism, and were not taking any drug known to affect bone turnover. All subjects provided full informed consent, and the study was approved by the Mayo Clinic Institutional Review Board. Details regarding ensuring vitamin D sufficiency in these study subjects also have been described previously.(10)

At the time of entry into the study, the subjects were administered a long-acting gonadotropin-releasing hormone (GnRH) agonist [leuprolide acetate (Lupron-Depot), TAP Pharmaceuticals, Deerfield, IL, USA], 7.5 mg intramuscularly, to suppress endogenous T and E production. They also were started on the aromatase inhibitor [letrozole (Femara), Novartis], 2.5 mg/day. Physiologic T and E_2_ levels were maintained by starting the subjects on a T gel (AndroGel, Solvay Pharmaceuticals, Marietta, GA, USA), 5 g/day (delivering 5 mg/day of T), as well as an E_2_ patch (VivelleDot, Novartis), 37.5 µg/day. After 3 weeks following GnRH agonist administration and while maintaining letrozole, T, and E_2_ treatment, the subjects were admitted to the Mayo Clinical Research Unit (CRU) for their baseline visit. After an overnight fast, serum samples were drawn at 8 a.m. for the study assays. Following the baseline studies, the subjects were randomized into one of four groups: group A (–T, –E; *n* = 15) discontinued both T and E replacement, group B (–T, +E; *n* = 15) discontinued the T gel but continued the E patch, group C (+T, –E; *n* = 15) discontinued the E patch but continued the T gel, and Group D (+T, +E, *n* = 14) continued both the T gel and the E patch. All subjects received a second dose of the GnRH agonist, and all subjects continued letrozole treatment throughout the study period. Three weeks following randomization, the subjects were readmitted to the CRU for their final visit, and repeat fasting (8 a.m.) blood samples were obtained. As for study A, serum samples were frozen and stored at –80°C, and only previously unthawed samples were used in all analyses in this study.

### Hormonal and biochemical assays

Serum calcium and phosphorus concentrations were measured by an automated photometric assay [interassay coefficient of variation (CV) < 10%; Roche Diagnostics, Madison, WI, USA]. Serum creatinine was measured using an automated enzymatic colorimetric assay (interassay CV < 10%; Roche Diagnostics). Serum 25-hydroxyvitamin D [25(OH)D] was measured using tandem mass spectroscopy (interassay CV < 7%; API 5000, Applied Biosystems-MDS Sciex, Carlsbad, CA, USA). Serum P1NP was measured by radioimmunassay [interassay CV < 10%; Immunodiagnostic Systems (IDS, Scotsdale, AZ, USA)], and serum osteocalcin (OCN) was measured using a two-site immunoradiometric assay (interassay CV < 8%; CIS-US, Bedford, MA, USA). Serum C-terminal telopeptide of type I collagen (CTX) was measured by ELISA (interassay CV < 8%; IDS), and serum tartrate-resistant acid phosphatase isoform type 5b (TRACP5b) also was measured by ELISA (interassay CV < 14%; IDS), as was serum osteoprotegerin (OPG; interassay CV < 8%; ALPCO Immunoassays, Salem, NH, USA).

To measure serum sclerostin levels, we used two different immunoassays in study A, an in-house assay and a commercial assay. Because we observed similar changes with the two assays, only the commercial assay was used in study B. For the in-house assay, we used an ELISA developed in our laboratory. A biotinylated goat antihuman sclerostin antibody (R&D Systems, Minneapolis, MN, USA) was added to streptavidin-coated plates followed by the samples and standards (recombinant human sclerostin, R&D Systems). After a 3-hour incubation, a monoclonal antihuman sclerostin antibody (R&D Systems) was added. Following a 1-hour incubation, antimouse IgG–horseradish peroxidase was added for 30 minutes. The plate was washed for a final time, substrate was added for 30 minutes, and the plate was read at 450/620. The interassay CV was 8%, and the lower limit of detection was 100 pg/mL. For the commercial assay, we used a recently available quantitative sandwich ELISA obtained from ALPCO (developed by Biomedica, Vienna, Austria).(22) The interassay CV was 4%, and the lower limit of detection was 86 pg/mL.

### Statistical analysis

For study A, the comparisons between the control and E-treated women were made using two-sample, two-sided *t* tests. For study B, as described previously,(8,10) the primary method of analysis to dissect out effects of E versus T on the various parameters took advantage of the factorial design. Thus we used a two-factor ANOVA model to compare the changes in the variables in the +E (groups B and D) versus–E (groups A and C) and +T (groups C and D) versus–T (groups A and C) groups (essentially pooling the two –E and two +E groups for comparison and similarly for the –T and +T groups). For both studies, the relationships between various variables of interest were defined using the Pearson correlation. A one-sample, two-sided *t* test was used to assess percent changes from baseline for variables where we had baseline and final measurements. Results were considered significant at the *p* < .05 level.

## Results

### Baseline parameters in study subjects

Table 1 shows the baseline anthropometric and biochemical variables in the study subjects. As is evident, for both studies, the subjects in the various groups were well matched for all these variables. Similarly, bone turnover markers and serum sclerostin levels (measured using both the in-house and commercial assays in the women but only the commercial assay in the men) were similar across all study groups at baseline (Table 2).

**Table 1:** Baseline Anthropometric and Biochemical Parameters in the Study Subjects

Study A: Women			

Group	Control	E	*p* Value^a^
*N*	18	17	
Age, years	54 ± 1	54 ± 1	.902
Height, m	1.67 ± 0.01	1.63 ± 0.02	.047
Weight, kg	76.2 ± 3.7	74.7 ± 3.0	.762
Body mass index (BMI), kg/m^2^	27.4 ± 1.3	28.3 ± 1.3	.592
Serum parameters			
Ca, mg/dL	9.7 ± 0.1	9.7 ± 0.1	.692
Phosphorus, mg/dL	3.7 ± 0.1	3.9 ± 0.1	.335
Cr, mg/dL	1.0 ± 0.03	0.9 ± 0.03	.204
25(OH)D, ng/mL	31.3 ± 2.1	33.5 ± 1.6	.425

Study B: Men					

Group	–T, –E	–T, +E	+T, –E	+T, +E	*p* Value^b^
*N*	15	15	15	14	
Age, years	66 ± 3	66 ± 2	70 ± 1	66 ± 3	0.603
Height, m	1.76 ± 0.01	1.77 ± 0.03	1.79 ± 0.02	1.79 ± 0.02	0.701
Weight, kg	87.3 ± 2.9	86.7 ± 3.4	88.4 ± 2.3	90.0 ± 3.3	0.879
BMI, kg/m^2^	28.1 ± 1.0	27.7 ± 0.9	27.7 ± 0.7	28.1 ± 0.9	.959
Serum parameters					
Ca, mg/dL	9.4 ± 0.1	9.6 ± 0.1	9.4 ± 0.1	9.4 ± 0.1	.318
Phosphorous, mg/dL	3.4 ± 0.1	3.5 ± 0.1	3.3 ± 0.1	3.1 ± 0.1	.128
Cr, mg/dL	1.1 ± 0.03	1.2 ± 0.04	1.1 ± 0.03	1.2 ± 0.03	.189
25(OH)D, ng/mL	31.1 ± 1.6	28.3 ± 2.2	32.1 ± 2.5	32.2 ± 2.5	.579

a For comparison of control and estrogen groups.

b For comparison of all four groups by ANOVA.

**Table 2:** Baseline Bone Turnover Markers and Sclerostin Levels in the Study Subjects

Study A: Women			

Group	Control	E	*p* Value^a^
P1NP, µg/L	47.9 ± 3.2	58.3 ± 7.0	.174
OCN, ng/mL	29.8 ± 2.2	28.8 ± 2.1	.745
CTX, ng/mL	0.73 ± 0.06	0.74 ± 0.08	.936
TRACP5b, U/L	4.0 ± 0.3	4.2 ± 0.3	.716
Sclerostin (in-house assay), pg/mL	579 ± 67	555 ± 55	.782
Sclerostin (commercial assay), pg/mL	312 ± 21	302 ± 19	.731

Study B: Men					

Group	–T, –E	–T, +E	+T, –E	+T, +E	*p* Value^b^
P1NP, µg/L	38.6 ± 3.2	43.5 ± 5.5	40.4 ± 3.2	41.9 ± 5.5	.880
CTX, ng/mL	0.54 ± 0.05	0.55 ± 0.8	0.50 ± 0.05	0.54 ± 0.07	.940
TRACP5b, U/L	3.8 ± 0.3	2.9 ± 0.2	3.4 ± 0.3	3.1 ± 0.3	.085
Sclerostin (commercial assay), pg/mL	439 ± 33	446 ± 44	406 ± 29	447 ± 47	.861

a For comparison of control and estrogen groups.

b For comparison of all four groups by ANOVA.

### Changes in bone turnover markers and in sclerostin levels following hormonal manipulations

**Fig. 1:**
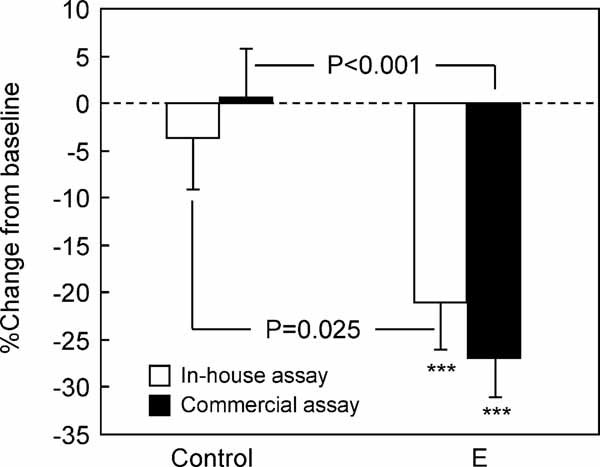
Percent change from baseline in serum sclerostin levels in the subjects in study A using either the in-house assay (*open bars*) or the commercial assay (*solid bars*). *p* Values for differences in changes in the control versus E-treated groups are as indicated. ***p < .001 for significance of change from baseline.

**Fig. 2:**
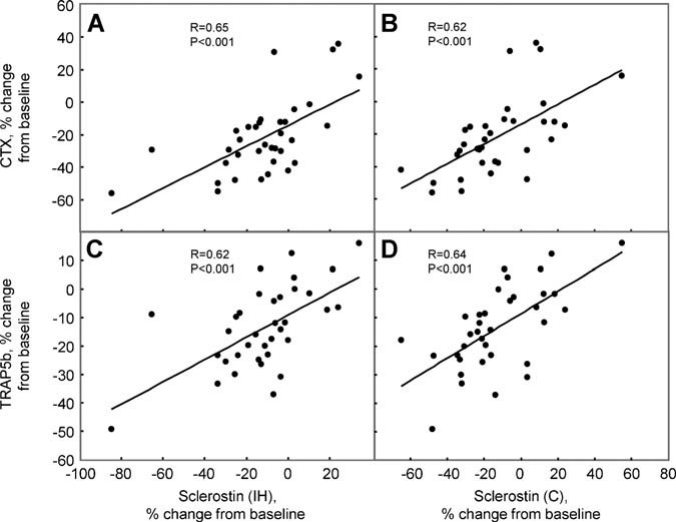
Correlations between percent changes in serum CTX in the two groups of women in study A combined versus percent change in serum sclerostin levels using either the in-house (*A*) or commercial (*B*) assay. Panels *C* and *D* show the analogous relationships for TRACP5b versus sclerostin using either the in-house assay or commercial assay, respectively.

Table 3 shows the percent changes from baseline in bone turnover markers in the study subjects. As has been described before for the early effects of E treatment on bone-formation markers,(5) 4 weeks of E treatment in the women (study A) led to significant increases in bone-formation markers (P1NP and OCN) and decreases in bone-resorption markers (CTX and TRACP5b). As shown in Fig. 1, these changes were accompanied by significant reductions in serum sclerostin levels using both the in-house and commercial assays. Using data from both groups combined, the percent change in serum sclerostin levels using the in-house assay were significantly correlated with the percent changes in serum sclerostin levels using the commercial assay (*r* = 0.76, *p* < .0001). However, the percent changes in serum P1NP or OCN levels did not correlate with percent changes in sclerostin levels using either assay (*r* = 0.15, *p* = .410, and *r* = –0.08, *p* = .649 for P1NP and OCN, respectively, for the in-house assay, and *r* = –0.06, *p* = .754, and *r* = –0.10, *p* = .563, for P1NP and OCN, respectively, for the commercial assay). Of interest, however, the percent change in serum CTX did correlate significantly with changes in sclerostin levels using either the in-house (Fig. 2*A*) or commercial assay (Fig. 2*B*), with similar correlations for TRACP5b (Fig. 2*C, D*).

**Table 3:** Percent Changes from Baseline in Bone Turnover Markers in the Study Subjects Following the Hormonal Manipulations

Study A: Women			

Group	Control	E	*p* Value^a^
P1NP	12.2 ± 6.4	42.1 ± 9.3***	.012
OCN	−2.0 ± 2.7	6.6 ± 2.8*	.034
CTX	−7.5 ± 5.2	−36.8 ± 3.0***	<.001
TRACP5b	−2.6 ± 2.1	−25.3 ± 2.2***	<.001

Study B: Men						

					*p* Value^b^	
					
Group	–T, –E	–T, +E	+T, –E	+T, +E	E effect	T effect
P1NP	−13.4 ± 4.9*	−8.3 ± 2.3**	−17.4 ± 2.9***	−3.7 ± 2.9	.015	.486
CTX	56.3 ± 14.3**	14.2 ± 7.3	53.3 ± 14.1**	10.2 ± 7.9	<.001	.760
TRACP5b	16.7 ± 2.4***	5.9 ± 3.1	17.2 ± 3.4***	2.6 ± 1.5	<.001	.614

*Note:* For each group (eg, Men, –T, –E), the baseline values for each marker for that group, as shown in Table 2, were used to calculate percent changes.

* *p* < .05

** *p* < .01

*** *p* < .001 versus baseline.

a *p* Value for comparison of the change in the E versus the control groups.

b *p* Value using a two-factor ANOVA model for E or T effects.

**Fig. 3:**
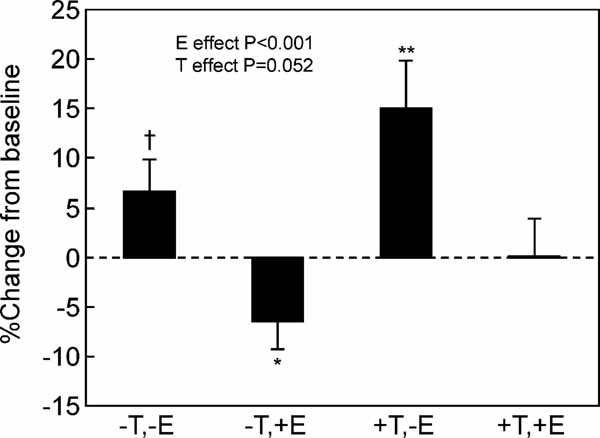
Percent change from baseline in serum sclerostin levels in the subjects in study B. The *p* values for the E and T effects are based on the two-factor ANOVA model described under “Methods.” Briefly, this compares changes in the +E versus –E groups for the E effect and changes in the +T versus the –T groups for the T effect. *p < .05, **p < .01, and ^†^*p* = .051 for significance of change from baseline.

Table 3 also shows the changes in bone turnover markers in the men (study B) following their hormonal manipulations. As reported previously(8,10) with acute sex steroid deficiency (–T, –E), serum P1NP levels decreased significantly in the men, and this decrease was attenuated by E, but not by T, treatment. Conversely, bone-resorption markers increased significantly following sex steroid deficiency, and these increases were prevented by E, but not by T, treatment. Given that the in-house and commercial sclerostin assays performed very similarly in the women (Fig. 1), we only used the commercial assay in men, and Fig. 3 shows the percent changes from baseline in serum sclerostin levels in the four groups of men. As is evident, serum sclerostin levels increased following sex steroid deficiency in the –T, –E group; decreased significantly in the –T, +E group; increased significantly in the +T, –E group; and did not change in the sex steroid–sufficient group (+T, +E). Using the two-factor ANOVA model, there was a highly significant E effect (*p* < .001) on reducing sclerostin levels and a borderline significant (*p* = .052) T effect on increasing sclerostin levels, suggesting opposite effects of E versus T on sclerostin levels. Similar to the findings in the women, percent changes in serum P1NP levels in the four groups combined did not correlate with changes in sclerostin levels (*r* = 0.05, *p* = .693). However, as in the women, changes in serum CTX and in TRACP5b did correlate significantly with changes in sclerostin levels (*r* = 0.33, *p* = .009, and *r* = 0.32, *p* = .014, for CTX and TRACP5b, respectively).

Since previous studies have demonstrated that *OPG* is a Wnt target gene,(23) we tested whether the observed correlations between changes in serum sclerostin levels and bone resorption markers were accompanied by parallel changes in serum OPG levels. Table 4 shows the baseline OPG levels in the subjects in the two studies, as well as changes following the hormonal manipulations. As is evident, there were no significant changes in serum OPG levels in any of the groups. Moreover, changes in sclerostin levels did not correlate with changes in OPG levels either in study A or study B (data not shown).

**Table 4:** Baseline and Percent Changes in Serum OPG Levels in the Study Subjects Following the Hormonal Manipulations

Study A: Women			

Group	Control	E	*p* Value^a^
OPG, pmol/L	3.4 ± 0.4	3.0 ± 0.3	.463
OPG, % change	−1.6 ± 4.5	−6.7 ± 5.0	.448

Study B: Men						

Group	–T, –E	–T, +E	+T, –E	+T, +E	*p* Value^b^	
OPG, pmol/L	2.9 ± 0.2	2.9 ± 0.2	2.6 ± 0.1	2.8 ± 0.3	0.624	

					E effect	T effect
OPG, % change	3.8 ± 3.5	1.6 ± 5.3	−2.8 ± 3.3	−2.1 ± 3.7	.842	.207

*Note:* For each group, the percent changes were calculated using the respective baseline values.

* *p* < .05; **p < .01; ***p < .001 versus baseline.

a *p* Value for comparison of baseline values or the percent change in the E versus the control groups.

b *p* Value for comparison of four groups at baseline by ANOVA or for E or T effects using a two-factor ANOVA model.

## Discussion

Using two different study designs (E treatment versus E withdrawal) and in both women and men, we demonstrate in this study that E treatment reduces, whereas E withdrawal increases, serum sclerostin levels. These data clearly establish that circulating sclerostin levels and, presumably, sclerostin production by osteocytes in bone are regulated by E. Our findings are consistent with the recent observation by Mirza and colleagues(20) that serum sclerostin levels are inversely associated with the free E_2_ index in postmenopausal women. However, using direct interventions, our data provide proof of causality, which only can be inferred by correlations. While our studies do not establish whether E regulates sclerostin production directly or indirectly, Huang and colleagues(24) recently have identified a –9247 T/C polymorphism in the *SOST* upstream regulatory region that is associated with spine, femoral neck, trochanter, and total hip bone mineral density (BMD) and is located at the core consensus recognition site of two cooperating transcription factors, C/EBPα and FOXA1, that modulate E receptor (ER) function. Thus C/EBPα has been shown to interact with ERα in glutathione-*S*-transferase pull-down experiments,(25) and knock-down of *FOXA1* expression has been shown to block the association of the ER with chromatin and expression of E-induced genes.(26,27) Moreover, the *SOST* promoter has three additional classical E-response elements between the –9247 polymorphism and the transcriptional start site.(24) As such, the *SOST* gene may be a direct target for E, but clearly, further studies are needed to address whether E regulates *SOST* expression at a transcriptional level.

In contrast to E, our data in study B indicate that T (in the absence of aromatization to E) fails to suppress, and appears to increase, circulating sclerostin levels. While T previously has been thought of as being “anabolic” for bone, direct evidence for this is relatively sparse.(2) On the contrary, males with mutations either in the *ERα* gene(28,29) or in the gene encoding aromatase(30,31) have marked deficits in bone mass despite having normal or elevated T levels. The biologic significance of the apparent stimulatory effects of T on circulating sclerostin levels, however, needs to be defined by additional studies in humans and animal models.

In study A, we used two independent sclerostin assays to evaluate changes following E treatment, with virtually identical results. The fact that we got the same results using independently generated antibodies to sclerostin (R&D Systems and Biomedica) provides further confidence that our observations are valid independent of the sclerostin antibody used in the assays. However, since the commercial assay is easier to perform and has greater precision than our in-house assay, we used the commercial assay in study B.

Perhaps somewhat surprising was our finding that changes in sclerostin levels in both studies failed to correlate with changes in bone-formation markers but did correlate with changes in bone-resorption markers. The lack of correlation with the bone-formation markers could be due to several reasons: First, changes in sclerostin may account for only part of the effects of E in maintaining bone formation, given the known multiple pathways regulated by E that could have an impact on bone formation.(2,32) In addition, it is possible that changes in sclerostin production occur well before changes in bone-formation markers, and even sampling sclerostin levels 3 to 4 weeks following E treatment or withdrawal may have missed more robust changes in serum sclerostin levels early after changes in E status, which also might have shown stronger associations with the bone-formation markers. It is also possible that changes in peripheral sclerostin levels may not fully reflect alterations in sclerostin production in the bone microenvironment; however, we have found recently (33) that circulating sclerostin levels are highly correlated with bone marrow plasma sclerostin levels, suggesting that peripheral sclerostin levels likely do accurately reflect sclerostin levels in the bone microenvironment.

In contrast to the bone-formation markers, changes in circulating sclerostin levels did correlate with changes in the bone-resorption markers. These findings are consistent with a recent study in rats showing that treatment of ovariectomized rats with a neutralizing antibody to sclerostin not only increased bone-formation rates but also reduced osteoclast numbers on bone surfaces.(19) Similarly, treatment of postmenopausal women for up to 85 days with an antisclerostin antibody also resulted in an increase in bone-formation markers as well as a decrease in bone-resorption markers.(34) These data in rats and humans are entirely consistent with our findings, and increasing evidence now indicates that changes in sclerostin activity regulate not only bone formation but also bone resorption. Since *OPG* is a known Wnt target gene,(23) we also tested whether the association between changes in sclerostin levels and changes in bone-resorption markers could be explained by alterations in circulating OPG levels. However, this was not the case, and these findings would suggest that mechanisms other than regulation of OPG may account for the relationship between sclerostin levels and bone-resorption markers, although we cannot exclude the possibility that the lack of changes in serum OPG levels were related to our relatively small sample size and/or the fact that OPG is produced by a number of nonskeletal tissues.(35) As such, serum OPG levels may not reflect changes in the bone microenvironment. Nonetheless, it is of interest that Wnt signaling has been implicated recently in regulating not only bone formation but also bone resorption. Thus activation of Wnt signaling has been shown to downregulate *RANKL* mRNA and protein expression in osteoblasts(36) and to directly inhibit the differentiation of osteoclast precursor cells through the canonical pathway.(37,38)

While our findings are consistent with E regulation of sclerostin production in humans, we recognize that there is currently no information on the stability of sclerostin or its degradation in the circulation. Thus we cannot exclude an effect of E on the metabolism of sclerostin; further studies addressing this issue and, as noted earlier, examining possible direct transcriptional regulation of the *SOST* gene by E need to be done.

In summary, our studies demonstrate that in both sexes, E decreases circulating sclerostin levels. While the reduction in sclerostin production may play a role in the effects of E in maintaining bone formation, the associations we observed between changes in sclerostin levels and in bone-resorption markers suggest that at least part of the antiresorptive effects of E also may be mediated via changes in sclerostin production. Our findings thus identify sclerostin as a potentially important mediator of E effects on bone turnover, and further human and animal studies are needed to more precisely define how much of the overall E effect on bone formation and resorption can be explained by changes in sclerostin production.

## References

[1] Lufkin EG, Wahner HW, O'Fallon WM (1992). Treatment of postmenopausal osteoporosis with transdermal estrogen. Ann Intern Med..

[2] Riggs BL, Khosla S, Melton LJ (2002). Sex steroids and the construction and conservation of the adult skeleton. Endocr Rev..

[3] Jilka RL, Takahashi K, Munshi M, Williams DC, Roberson PK, Manolagas SC (1998). Loss of estrogen upregulates osteoblastogenesis in the murine bone marrow. J Clin Invest..

[4] Charatcharoenwitthaya N, Khosla S, Atkinson EJ, McCready LK, Riggs BL (2007). Effect of blockade of TNF-α and interleukin 1 action on bone resorption in early postmenopausal women. J Bone Miner Res..

[5] Hannon R, Blumsohn A, Naylor K, Eastell R (1998). Response of biochemical markers of bone turnover to hormone replacement therapy: impact of biological variability. J Bone Miner Res..

[6] Lean JM, Chow JW, Chambers TJ (1994). The rate of cancellous bone formation falls immediately after ovariectomy in the rat. J Endocrinol..

[7] Manolagas SC (2000). Birth and death of bone cells: basic regulatory mechanisms and implications for the pathogenesis and treatment of osteoporosis. Endocr Rev..

[8] Falahati-Nini A, Riggs BL, Atkinson EJ, O'Fallon WM, Eastell R, Khosla S (2000). Relative contributions of testosterone and estrogen in regulating bone resorption and formation in normal elderly men. J Clin Invest..

[9] Leder BZ, LeBlanc KM, Schoenfeld DA, Eastell R, Finkelstein JS (2003). Differential effects of androgens and estrogens on bone turnover in normal men. J Clin Endocrinol Metab..

[10] Sanyal A, Hoey KA, Modder UI (2008). Regulation of bone turnover by sex steroids in men. J Bone Miner Res..

[11] Baron R, Rawadi G (2007). Targeting the Wnt/beta-catenin pathway to regulate bone formation in the adult skeleton. Endocrinology..

[12] Krishnan V, Bryant HU, MacDougald OA (2006). Regulation of bone mass by Wnt signaling. J Clin Invest..

[13] Armstrong VJ, Muzylak M, Sunters A (2007). WNT/B-Catenin signaling is a component of osteoblastic bone cells' early responses to load-bearing, and requires estrogen receptor a. J Biol Chem..

[14] Brunkow ME, Gardner JC, Van Ness J (2001). Bone dysplasia sclerosteosis results from loss of the SOST gene product, a novel cystine knot-containing protein. Am J Hum Genet..

[15] Balemans W, Ebeling M, Patel N (2001). Increase bone density in sclerosteosis is due to the deficiency of a novel secreted protein (SOST). Hum Mol Genet..

[16] Staehling-Hampton K, Proll S, Paeper BW (2002). A 52-kb deletion in the SOST-MEOX1 intergenic region on 17q12-q21 is associated with van Buchem disease in the Dutch population. Am J Med Genet..

[17] Balemans W, Patel N, Ebeling M (2002). Identification of a 52kb deletion downstream of the SOST gene in patients with van Buchem disease. J Med Genet..

[18] Li X, Ominsky MS, Niu Q-T (2008). Targeted deletion of the sclerostin gene in mice results in increased bone formation and bone strength. J Bone Miner Res..

[19] Li X, Ominsky MS, Warmington KS (2009). Sclerostin antibody treatment increases bone formation, bone mass, and bone strength in a rat model of postmenopausal osteoporosis. J Bone Miner Res..

[20] Mirza FS, Padhi ID, Raisz LG, Lorenzo JA (2010). Serum sclerostin levels negatively correlate with parathyroid hormone levels and free estrogen index in postmenopausal women. J Clin Endocrinol Metab..

[21] Clowes JA, Eghbali-Fatourechi GZ, McCready L, Oursler MJ, Khosla S, Riggs BL (2009). Estrogen action on bone marrow osteoclast lineage cells of postmenopausal women in vivo. Osteoporos Int..

[22] Terpos E, Christoulas D, Katodritou E (2009). High serum sclerostin correlates with advanced stage, increased bone resorption, reducted osteoblast function, and poor survival in newly-diagnosed patients with multiple myeloma. Blood..

[23] Glass DA, Bialek P, Ahn JD (2005). Canonical Wnt signaling in differentiated osteoblasts controls osteoclast differentiation. Dev Cell..

[24] Huang Q-Y, Li GHY, Kung AWC (2009). The -9247 T/C polymorphism in the SOST upstream regulatory region that potentially affects C/EBPalpha and FOXA1 binding is associated with osteoporosis. Bone..

[25] Boruk M, Savory JG, Hache RJ (1998). AF-2-dependent potentiation of CCAAT enhancer binding protein beta-mediated transcriptional activation by glucocorticoid receptor. Mol Endocrinol..

[26] Laganiere J, Deblois G, Lefebvre C, Bataille AR, Robert F, Giguere V (2005). Location analysis of estrogen receptor alpha target promoters reveals that FOXA1 defines a domain of the estrogen response. Proc Natl Acad Sci USA..

[27] Carroll JS, Liu S, Brodsky AS (2005). Chromosome-wide mapping of estrogen receptor binding reveals long-range regulation requiring the forkhead protein FoxA1. Cell..

[28] Smith EP, Boyd J, Frank GR (1994). Estrogen resistance caused by a mutation in the estrogen-receptor gene in a man. N Engl J Med..

[29] Smith EP, Specker B, Bachrach BE (2008). Impact on bone of an estrogen receptor-alpha gene loss of function mutation. J Clin Endocrinol Metab..

[30] Carani C, Qin K, Simoni M (1997). Effect of testosterone and estradiol in a man with aromatase deficiency. N Engl J Med..

[31] Bilezikian JP, Morishima A, Bell J, Grumbach MM (1998). Increased bone mass as a result of estrogen therapy in a man with aromatase deficiency. N Engl J Med..

[32] Syed F, Khosla S (2005). Mechanisms of sex steroid effects on bone. Biochem Biophys Res Commun..

[33] Drake MT, Srinivasan B, Mödder UI (2010). Effects of parathyroid hormone treatment on circulating sclerostin levels in postmenopausal women. J Clin Endocrinol Metab..

[34] Padhi D, Stouch B, Jang G (2007). Anti-sclerostin antibody increases markers of bone formation in healthy postmenopausal women. American Society for Bone and Mineral Research Annual Meeting..

[35] Kearns AE, Khosla S, Kostenuik PJ (2008). Receptor activator of nuclear factor kappaB ligand and osteoprotegerin regulation of bone remodeling in health and disease. Endocr Rev..

[36] Spencer GJ, Utting JC, Etheridge SL, Arnett TR, Genever PG (2006). Wnt signalling in osteoblasts regulates expression of the receptor activator of NFkappB ligand and inhibits osteoclastogenesis in vitro. J Cell Sci..

[37] Modarresi R, Xiang Z, Yin M, Laurence J (2009). WNT/beta-catenin signaling is involved in regulation of osteoclast differentiation by human immunodeficiency virus protease inhibitor ritonavir: relationship to human immunodeficiency virus-linked bone mineral loss. Am J Pathol..

[38] Qiang Y-W, Chen Y, Brown N (2009). Characterization of Wnt/beta-catenin signaling in osteoclasts in multiple myeloma. Br J Haematol..

